# Beyond Reasonable Doubt: Evolution from DNA Sequences

**DOI:** 10.1371/journal.pone.0069924

**Published:** 2013-08-08

**Authors:** W. Timothy J. White, Bojian Zhong, David Penny

**Affiliations:** 1 Institute of Fundamental Sciences, Massey University, Palmerston North, New Zealand; George Washington University, United States of America

## Abstract

We demonstrate quantitatively that, as predicted by evolutionary theory, sequences of homologous proteins from different species converge as we go further and further back in time. The converse, a non-evolutionary model can be expressed as probabilities, and the test works for chloroplast, nuclear and mitochondrial sequences, as well as for sequences that diverged at different time depths. Even on our conservative test, the probability that chance could produce the observed levels of ancestral convergence for just one of the eight datasets of 51 proteins is ≈1×10^−19^ and combined over 8 datasets is ≈1×10^−132^. By comparison, there are about 10^80^ protons in the universe, hence the probability that the sequences could have been produced by a process involving unrelated ancestral sequences is about 10^50^ lower than picking, among all protons, the same proton at random twice in a row. A non-evolutionary control model shows no convergence, and only a small number of parameters are required to account for the observations. It is time that that researchers insisted that doubters put up testable alternatives to evolution.

## Introduction

There are some areas of science where there is still strong resistance to basic scientific conclusions: anthropogenic climate change [1], the reality of long term evolution [2] http://www.dissentfromdarwin.org, the origin of life, and the safety and efficacy of vaccination programs [3] are well-known examples. Thus we still require strong quantitative tests of our main scientific hypotheses, even if the conclusions appear obvious to most researchers. In the case of evolution, a strong prediction of Darwin’s ‘descent with modification’ [4] is that, as we go further and further back in time, the sequences for a given protein should become increasingly similar – we call this either ‘ancestral convergence’ or ‘reverse convergence’. The prediction from evolutionary theory is that DNA or protein sequences carrying out the same basic functions in different organisms are generally inherited from a common ancestor – in this sense they are fully homologous proteins (or orthologs) [5]. We must be able to measure this convergence and test it quantitatively. In practice, although the information comes primarily from DNA sequences, we convert them to protein sequences for the tests. As we see later, we currently cannot yet find any other hypothesis that leads inevitably to the same prediction without an explosive increase in the number of parameters.

It is basic to science that we have never tested all possible hypotheses; consequently we never obtain final and absolute knowledge about any aspect of the universe. Nevertheless, the scientific method provides us with the best form of knowledge that humans can attain, and ensures that we use the most thoroughly tested understanding at any time [6]. This Popperian framework allows both Bayesian and frequentist approaches to be used, dependent on what is appropriate for the questions being tested.

We use a non-evolutionary null model and develop a quantitative test of ancestral convergence, and apply it to a range of datasets that have diverged at deeper and deeper times. As a control we show that unrelated proteins do not show convergence. Furthermore, an excessive number of free parameters are required to account for the observed convergence by other processes. This clearly does not ‘prove’ that yet unknown models are impossible, but the theory of evolution leads to extremely strong predictions, and so the onus is now on others to propose testable alternatives.

## Materials and Methods

**Figure 1:**
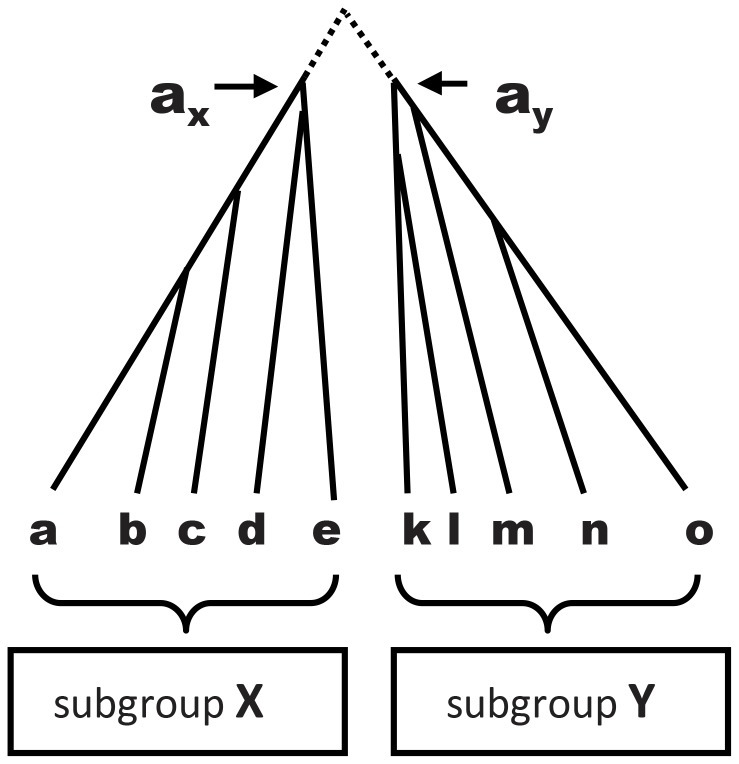
We use two natural subgroups (X and Y), independently align the sequences for the species in each subgroup, independently determine the optimal tree for each subgroup, independently infer the ancestral sequences a_x_ and a_y_ on the optimal subtrees (in practice the sequence at the nearest node to the root of the subtree is estimated), and finally measure the pairwise alignment score between the ancestral sequences, s(a_x_,a_y_). Separately, we measure the alignment score between each pair of sequences (s(_i,j_)) with one member in each of the two subsets, for example, s(_a,k_), s(_a,l_), s(_a,m_), and so on.

We develop a statistical test for quantifying convergence that consists of eight simple steps For Step 1 we take two subgroups of taxa X and Y (see Figure 1) that on independent evidence have non-overlapping subtrees; that is, they are natural subgroups (or clades). For example, with chloroplast sequences, we select subgroups based on nuclear and/or mitochondrial data [7], [8], and only later check that the subgroups are also supported by the chloroplast sequences. For each subgroup we independently align the sequences (Step 2); infer a subtree (Step 3); and infer the ancestral sequences a_x_ and a_y_ for the deepest nodes of each subtree (Step 4). For this step we use PAML [9], which is a well-established method that is robust to small changes in the tree [10]. Our test is conservative in that ancestral sequences are estimated independently: information from subgroup X is not used to estimate the ancestral sequence for subgroup Y, nor vice versa. We used the cpREV model [11] for inferring chloroplast trees, the WAG model [12] for nuclear proteins, and the mtREV24 model [13] for animal mitochondria. We obviously can never know whether these are the best possible models for estimating convergence, but any better models are predicted to show even greater convergence.

**Figure 2:**
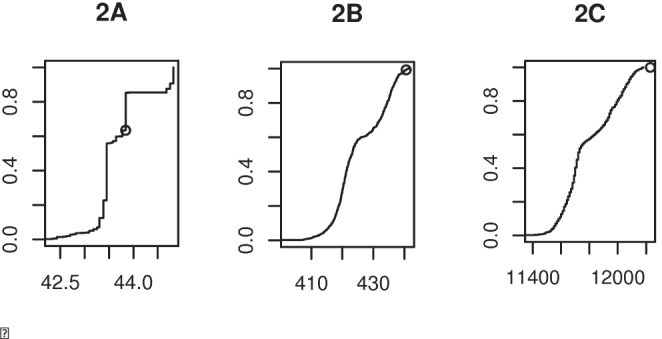
Cumulative frequency plots comparing the alignment score for the ancestral sequences (s(a_x,_a_y_), small circle) with the alignment scores of all pairs of proteins, s(*i*,*j*). The example is the monocot/eudicot chloroplast dataset and for the short protein psbF (**2A**), a longer protein atpA (**2B**), and the 51 concatenated genes (**2C**). The x-axis shows the alignment score, which increases with the length of the protein(s), and is largest for the 51 concatenated proteins. There are 1056 s(*i*,*j*) scores between pairs of 24 monocots and 44 eudicots, and the y-axis indicates where the s(a_x,_a_y_) fits as a proportion of this number. For some short proteins in particular, multiple s(*i*,*j*) values equal the ancestral score s(a_x_,a_y_), and in this case our test conservatively places the ancestral score below the rest (as in psbF in Fig 2A).

The program MUSCLE [14] is used for calculating alignment scores, see details later. For Step 5, the pairwise alignment score s(a_x_,a_y_) is then calculated between the inferred ancestral sequences a_x_ and a_y_ (we call this the ‘ancestral score’), with higher values showing that ancestral sequences are more similar (Table 1). In Step 6 we then calculate the alignment score s(*i*,*j*) for all pairs of sequences (with just one sequence from each of the two subgroups). From the resulting distribution of between-subgroup scores (see Figure 2) we calculate (Step 7) the probability *p* of observing scores at least as high as the ancestral score under the null model, which we now describe.

**Table 1:** Calculation of alignment score for the inferred ancestral monocot and eudicot sequences of the psbK gene.

1									10										20										30	
M	L	N	I	L	N	L	I	C	I	C	L	N	S	A	P	Y	S	S	S	F	F	C	A	K	–	–	P	A	Y	
M	L	N	I	I	S	L	–	–	I	C	L	N	S	A	L	H	S	S	S	F	F	F	A	K	L	P	E	A	Y	
1.010	0.771	0.840	0.759	0.376	0.328	0.771	−0.729	0.000	0.759	3.949	0.771	0.840	0.535	0.563	0.134	0.454	0.535	0.535	0.535	1.521	1.521	0.125	0.563	0.682	−0.725	0.000	0.207	0.563	1.877	
**31**									**40**										**50**										**60**	
A	V	F	N	P	I	V	D	F	M	P	V	I	P	V	L	F	F	L	L	A	F	V	W	Q	A	A	V	S	F	R
A	F	F	N	P	I	V	D	F	M	P	V	I	P	V	L	F	F	L	L	A	L	V	W	Q	A	A	V	S	F	R
0.563	0.228	1.521	0.840	1.812	0.759	0.664	1.047	1.521	1.010	1.812	0.664	0.759	1.812	0.664	0.771	1.521	1.521	0.771	0.771	0.563	0.376	0.664	7.849	0.717	0.563	0.563	0.664	0.535	1.521	1.072

The ancestral sequences for the monocots, and the eudicots, are inferred independently, and then the alignment scores calculated in the program MUSCLE. The individual column scores depend on the frequencies and properties of the two amino acids; higher scores are given for pairs that are similar (readily substitutable) or specific (more readily substitutable for each other than for other amino acids). The column scores are summed to produce the alignment score.

Our null model can be considered in the following way - that the taxa in subgroup X are descended from an unknown number 1< = r_X_< = |X| of root sequences, the taxa in subgroup Y are descended from an unknown number 1< = r_Y_< = |Y| of root sequences, and that the r_X_+r_Y_ root sequences are all independent from each other. This allows, at one end of the spectrum, the possibility that all |X|+|Y| taxa were independently created, and at the other end of the spectrum, the possibility that all taxa in one subgroup are descended from a single common ancestor *for that subgroup*, which was created independently of the single common ancestor for the other subgroup. In other words, this null model imposes no requirements on the presence or absence of internal (within-subgroup) evolution of the two subgroups of taxa; the only constraint is that there is no evolutionary link between the two subgroups. That is, neither subgroup contains taxa derived from the other, nor from a common ancestor.

That the numbers r_X_ and r_Y_ are not specified helps generalise the null model because a tree built on all taxa in X using any statistically consistent method will necessarily contain r_X_ long edges from some “central” node to the subtrees containing the taxa. (Sampling error will in general cause these long edges to be connected to the central node by one or more short edges, rather than being a “pure” star tree, but these edges can be made arbitrarily short by using enough characters.).

**Figure 3:**
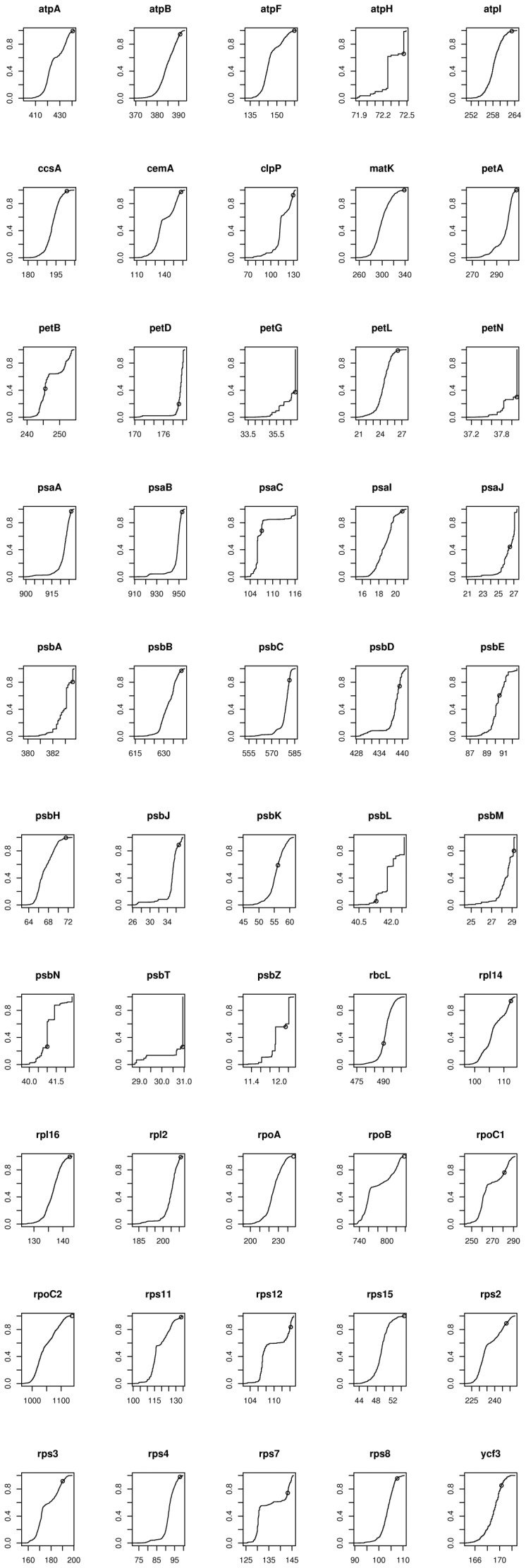
Cumulative distribution plots of the between-groups alignment scores for 50 of the 51 chloroplast proteins of monocots and eudicots (the plot for psbF is Fig 2A). The ancestral alignment score (s(*a_X_*,*a_Y_*)) is indicated by a small circle on each plot. There are 1056 comparisons (24 monocots×44 eudicots) for each protein. The y-axis is the same for each gene, but the x-axis is strongly dependent on the length of the protein (see also Figure 4).

Thus our non-evolutionary null model predicts that the similarity between the ancestral sequences is equal to the similarity between the extant sequences (that was calculated above in Step 7). When evolution from a common ancestor has occurred, the ancestral sequences will be significantly more similar than that predicted by the null model, and the null model will be rejected. (Some implications of the choice of null model are discussed further in the Discussion section.) For Step 8, additional power is achieved by using independent tests on different genes and combining the resulting *p* values [15] into a single value that represents the probability of observing data as, or more, extreme than that actually observed. Our test is again conservative: when handling between-group pairwise alignment scores equal to the ancestral score we consider these to be larger than the ancestral score (see Figure 3).

At this point we mention the possibility that two sister taxa could have been (mis-)placed in different subgroups. Although this does not fit within our null model, the only effect is to increase the measured between-subgroups average similarity, making it *harder* for the measured ancestral similarity to exceed it. Thus this model violation cannot induce a false positive (i.e. a claim that evolution is present when it is not) – only a false negative could occur. In any case, we aim to avoid these false negatives by selecting subgroups using external data.

It is important that our test can reject ancestral convergence with a control generated by a non-evolutionary process. This control differs only in that specific property for which we are testing: shared ancestry of homologous proteins for the subgroups X and Y. For this reason, each of our control datasets is a pair of subgroups of taxa X and Y as before, but in which the sequences used for subgroup X come from a different gene than those used for subgroup Y. This corresponds to (i.e. could be generated by) the “archetypes, followed by degeneration” model favoured by some pre-Darwinian biologists, discussed later. We do not expect to see convergence between, say, the ancestor of the monocot atpA gene and the ancestor of the eudicot psbF gene.

We measure the similarity of two sequences by the pairwise alignment score calculated using the MUSCLE alignment program [14] with default scoring parameters. The alignment score is the sum of the per-site scores, which are found from a pre-specified table that records the score for every possible combination of two amino acids, or one amino acid and a gap (see Table 1). The freedom in placing gaps means that different alignments of two given sequences are possible; the job of an alignment program such as MUSCLE is to find a high-scoring (ideally the highest-scoring) alignment. Note that setting the scores of all equal pairs of amino acids to 0 and all other scores to −1 will cause an alignment algorithm to recover an alignment having the fewest possible insertions, deletions and substitutions, and the number of these events (the *Levenshtein edit distance*) will be equal to the negative of the alignment score. This *edit* distance is a useful measure of similarity between strings that are not constrained to have the same length, but more biologically realistic alignments can be recovered by reducing the penalty for mutations between amino acids having similar codons or similar physical properties (e.g. size, hydrophobicity) as these mutations are more likely to occur or to survive into subsequent generations. Alignment quality is also improved by reducing the penalty incurred by multiple contiguous gap characters. MUSCLE’s default scoring parameters have been empirically tuned to work well with most protein datasets, and as such MUSCLE’s pairwise alignment score is a good measure of overall protein sequence similarity.

**Figure 4:**
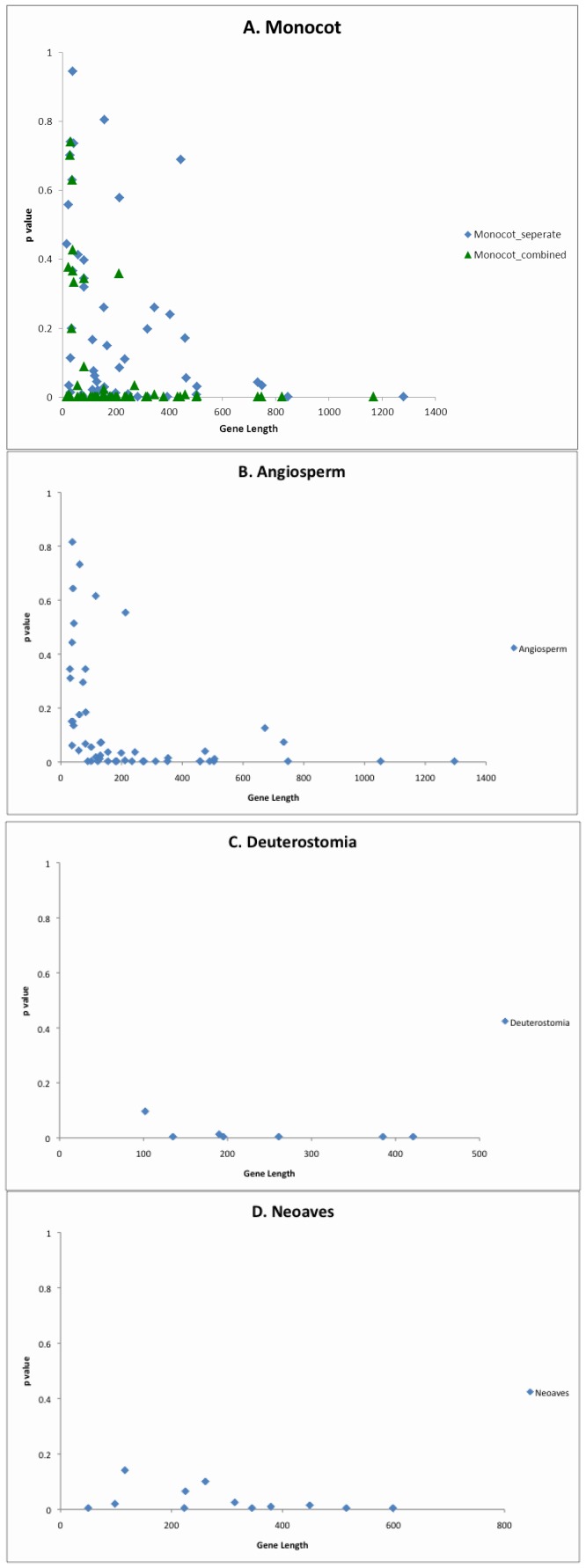
Protein length versus proportion of pairwise alignment scores higher than the ancestral score, for 4 datasets. Because of the possibility of slightly different gene lengths just one of the two datasets is used for illustration. As expected, longer proteins show convergence more strongly. Chloroplast results (4A and 4B) are for 51 chloroplast genes for divergences of monocots and for angiosperms (flowering plants). There are 7 nuclear proteins for 4C and 12 for the mitochondrial data in 4D.

Evolution is a stochastic process that involves reversals and parallel changes – for example, if the change val → ile is effectively neutral at a site, and has already occurred, then it is always possible that the reverse mutation (ile → val) will occur. For such reasons, the ancestral sequence actually inferred depends on a stochastic process, so although we do not expect the relation s(a_x,_a_y_)>s(*_i_,_j_*) to hold in every case we predict reliability increases as sequences become longer (Figure 4). This effect of sequence length is important support for the stochastic process of evolution, but that is not the primary focus here.

Fisher’s method [15] combines *p*-values from multiple independent tests of the same null hypothesis into a single *p*-value. We use it to combine the results of individual gene tests. Briefly, if the null hypothesis is true, then the *p*-value obtained from a test will be uniformly distributed between 0 and 1; taking the log and multiplying by −2 produces a quantity that is X^2^-distributed with 2 degrees of freedom. Thus the following statistic,
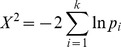
will be X^2^-distributed with 2*k* degrees of freedom. Once this statistic is calculated, a one-sided test can be used to extract a *p*-value from it, representing the probability of observing *k p*-values as low as those that were observed, assuming the null hypothesis (namely that the *k* original null hypotheses are correct).

The standard evolutionary model is relatively simple, and explains the basic tree-like structure of the sequences. To infer an ancestral sequence, the simplest models require only ≈190 parameters (a 20×20 symmetric matrix for the probability of changes between pairs of amino acids, less 20 because each row has to sum to 1). Then there is one additional parameter for each edge (branch) of the tree (there are 2*n*-3 edges for a binary tree, where *n* is the number of taxa). We could add one parameter for a probability of splitting of lineages, a second for an overall rate of change, a third for the distribution of rates across sites (e.g. for a Gamma distribution of rates), and a fourth for the proportion of invariable sites. Nevertheless as we later show, there are orders of magnitude fewer parameters required for a general evolutionary model than for a minimal ‘design’ model, and scientifically, we select the simpler model.

### Genuine Subgroups

It is important to demonstrate that the two subgroups or clades (X and Y) are genuine, and we do this for each of the subgroups in Table 2 in two ways. Firstly, the two subgroups are determined by other data – for example by nuclear or by mitochondrial DNA sequences for the plant chloroplast data. Secondly, for each of the eight pairs of datasets in Table 2 we later combine the two datasets, and confirm that the same two subgroups are still found – for example, the monocots and eudicots. This independent selection of the two subgroups is necessary because if, for example, we formed one subgroup by randomly selecting half the monocots and half the eudicot sequences, and used the other taxa to form the second subgroup, then we could artefactually get similar ancestors. So both tests (selecting subgroups from independent data, and later showing that the subgroups are recovered with the data used) are important in demonstrating that the subgroups X and Y are natural.

**Table 2:** Summary of X^2^ and p values for the different datasets.

Data type		Group X		Group Y	divergence times		X^2^	d.f.		p (Χ^2^)	Χ^2^ (control)	p (Χ^2^) (control)	
c/plast - 51	Eudicot (44)		Monocot (24)			∼125mya	289.0582	102	1.94E-19		93.68962	0.70943	
c/plast - 51	Angiosperm (25)		Gymnosperm (13)			∼305mya	363.5268	104	1.23E-29		85.64681	0.90482	
c/plast - 51	Seed plant (38)		Fern (7)			∼390mya	457.1184	102	1.69E-44		100.4507	0.52486	
c/plast - 51	Streptophyta (52)		Chlorophyta (6)			∼700mya	300.1617	94	2.23E-23		90.98169	0.56897	
Nuclear - 7	Vertebrata+Urochordata (9)		Echinoderms+Hemichords(10)			∼600mya	54.50034	14	1.05E-6		9.642275	0.78784	
Nuclear - 7	Deuterostomia (19)		Lophotrochozoa (12)			∼670mya	67.63153	14	5.17E-9		12.66025	0.55343	
mitochond-12	Neoaves (22)		Galloanserae (9)			∼80mya	99.55765	24	3.57E-11		16.03990	0.88663	
mitochond-12	Neognath (31)		Palaeognath (12)			∼100mya	102.5291	24	1.11E-11		23.69508	0.47914	
combined	all 8 pairs of datasets						1631.555	478	2.59E-132		432.8063	0.93172	
joint tree	Eudicot (44)		Monocot (24)			∼125mya	539.3154	102	1.51E-57				

The numbers of genes for the subgroups are indicated after the data type, and the number of taxa are indicated in parentheses ‘()’following the group name. The divergence times are minimum estimates from fossils and molecular data. Columns 5–7 relate to the probability that convergence could have arisen by chance, the last two columns are from controls where convergence is not expected. The penultimate row gives the combined values for the 8 datasets. The final row gives the results for the first example where combined information from both subsets (eudicots and monocots) is used for estimating the ancestral sequence of both subgroups; this again indicates that our test is very conservative.

### Estimating the Root of the Two Subtrees

There are several ways of estimating the root of the two subtrees, but in practice it appears to make little difference which of several methods we use. In the chloroplast example, the root of each subtree can be inferred from nuclear or mitochondrial DNA sequences (not chloroplast), and so is independent of the chloroplast data we use. This gives the position of the root in each subtree from prior information; alternatively they can be independently estimated by ‘midpoint rooting’. This can be done either by selecting the midpoint of the longest path, or the internal branch with the longest average of paths passing through it [16]. In practice, we take the node closest to the mid-point because we are estimating nodal sequences. There does appear to be an acceleration of the rate of evolution in the grasses [17], but, again in practice, this appeared to have little effect. The sequence of the root of the two subtrees appears to be quite robust.

Note that we could quite separately make an independent test for the similarity of evolutionary trees, by comparing the likelihood of chloroplast, nuclear, and mitochondrial datasets giving such highly similar trees. (Here we are only concerned, for example, about the ancestral sequences of the monocot/eudicot split – not the similarity of the trees as a whole.) Instead of computing alignment scores between pairs of sequences, maximum likelihood distances could in principle be computed for different-length sequences by using models of evolutionary change that allow for insertions and deletions, such as the TKF model – however software for computing these distances is apparently not currently available.

We start with chloroplast genomes because they have more than 50 protein genes (allowing both individual and combined tests); although there is some loss of genes from chloroplasts, there are no basic problems identifying homologous genes. In addition, there are several datasets at increasing levels of divergence, e.g.

monocots versus eudicots (both within flowering plants);

flowering plants angiospermsversus gymnosperms,

seed plants versus ferns and fern allies;

Streptophytes (land plants plus some green algae) versus Chlorophytes (most green algae).

These plant subsets have a wide range in their inferred divergence times; from about 125 to over 700 million years before the present [18], [19].

The tests are repeated on nuclear encoded sequences from animals, and then on avian mitochondrial genomes. With nuclear encoded proteins we test convergence for seven genes and for two groups for the deeper animal divergences, ranging from around 600–700 Mya [20]. The first test is for Vertebrata plus Urochordata versus Echinoderms plus Hemichordates. The second is for Deuterostomes versus Lophotrochozoa. For the nuclear datasets the 7 genes used are aldolase, methionine adenosyltransferase, ATP synthase beta chain, catalase, elongation factor 1 alpha, triosephosphate isomerase, and phosphofructokinase.

For mitochondrial sequences, we use a dataset from birds, using 12 protein-coding genes, and focus on two tests – firstly Neoaves [21] (most birds) versus Galloanseriforms (chickens and ducks), and secondly these two groups combined (neognaths) versus paleognaths (ratites and tinamous) [22]. Their estimated divergence times are around 80 and 100 million years ago, respectively [21], [22]. For the mitochondrial dataset, the 12 genes are ATP6, ATP8, COX1, COX2, COX3, Cytb, ND1, ND2, ND3, ND4, ND4L, and ND5.

## Results

Our primary results are very clear and are shown in Table 2. Our first example uses chloroplast genomes from 44 eudicotyledonous and 24 monocotyledonous flowering plants (monocots include grasses, palms and lilies). Combining results for all 51 genes gives a *p* value for our non-evolutionary null model of ≈2×10^−19^, shown in the top row of results in Table 2. This eudicot/monocot subdivision can be derived independently from either nuclear or mitochondrial DNA sequences [8], and so is independent of the chloroplast information. The 51 chloroplast proteins common to all lineages total 11,414 amino acids in length with an average length per protein of ∼225 amino acids (see Table 3). However, the proteins vary in length from 16 amino acids (psbZ) to 1168 amino acids (rpoC2). Across all 51 genes, on average 22% of pairwise scores were at least as high as the ancestral score, but this is mostly caused by a small number of shorter genes with relatively low ancestral scores (see Figure 3). Results are shown for each of the proteins in Table 3 and Figure 3. Figure 4 shows a correlation between protein sequence length and convergence, certainly consistent with a stochastic mechanism.

**Table 3:** Results for the 51 genes for the monocot/eudicot chloroplast dataset, and with the ancestral sequences (a_x_ and a_y_) inferred independently.

Gene	Gene length(amino acids)	Ancestral alignmentscore	Number of higher alignment scores	Proportion of higher alignment scores	Chi-squared term
atpA	503	440.6	8	0.0076	9.7656
atpB	433	390.7	58	0.0549	5.8036
atpF	178	159.9	2	0.0019	12.5382
atpH	81	72.5	364	0.3447	2.1302
atpI	241	263.2	10	0.0095	9.3193
ccsA	150	200.8	16	0.0152	8.3793
cemA	155	158.0	30	0.0284	7.1221
clpP	186	129.5	80	0.0758	5.1604
matK	380	338.8	1	0.0009	13.9245
petA	313	306.2	1	0.0009	13.9245
petB	213	245.6	611	0.5786	1.0943
petD	157	179.2	850	0.8049	0.4340
petG	37	36.8	665	0.6297	0.9249
petL	30	26.4	15	0.0142	8.5084
petN	29	38.1	741	0.7017	0.7085
psaA	748	926.2	35	0.0331	6.8138
psaB	733	952.9	45	0.0426	6.3112
psaC	81	107.1	338	0.3201	2.2784
psaI	24	20.8	35	0.0331	6.8138
psaJ	22	26.5	589	0.5578	1.1676
psbA	319	383.6	208	0.1970	3.2494
psbB	506	639.2	33	0.0313	6.9315
psbC	460	581.6	181	0.1714	3.5275
psbD	345	439.3	274	0.2595	2.6982
psbE	80	90.5	419	0.3968	1.8488
psbF	39	43.8	387	0.3665	2.0076
psbH	71	71.5	7	0.0066	10.0327
psbJ	30	36.6	119	0.1127	4.3662
psbK	57	56.2	436	0.4129	1.7692
psbL	38	41.3	998	0.9451	0.1130
psbM	34	29.2	211	0.1998	3.2208
psbN	43	41.0	778	0.7367	0.6110
psbT	30	30.9	782	0.7405	0.6008
psbZ	16	12.1	469	0.4441	1.6233
rbcL	443	490.1	728	0.6894	0.7439
rpl14	122	112.4	66	0.0625	5.5452
rpl16	126	142.4	8	0.0076	9.7656
rpl2	201	210.9	13	0.0123	8.7946
rpoA	256	246.2	0	0.0009	13.9245
rpoB	824	837.7	0	0.0009	13.9245
rpoC1	270	281.3	253	0.2396	2.8577
rpoC2	1168	1138.0	0	0.0009	13.9245
rps11	131	133.6	20	0.0189	7.9330
rps12	113	114.2	175	0.1657	3.5949
rps15	57	54.7	2	0.0019	12.5382
rps2	234	247.6	117	0.1108	4.4001
rps3	207	190.2	90	0.0852	4.9249
rps4	105	98.0	22	0.0208	7.7424
rps7	155	143.2	274	0.2595	2.6982
rps8	125	107.6	47	0.0445	6.2242
ycf3	157	170.3	158	0.1496	3.7993
Av/Sum	224.6	239.9	230.8	0.2186	289.0582

Our standard approach infers the ancestral sequences on the two subtrees independently. If we follow the more usual method and jointly infer the ancestral sequences on a single tree using the combined monocot and eudicot data we get, as expected, an even higher alignment score between the two ancestral sequences a_X_ and a_Y_, that is, the ancestral sequences are even more similar. Part of the reason for this is that we are using more information when inferring the ancestral sequences. Combining probabilities for all genes using Fisher’s method as before, we find that the probability of observing such high ancestral scores for the 51 chloroplast proteins under our non-evolutionary null model is 1.51×10^−57^ (compared with ≈2×10^−19^, see the top row of Table 2) - our test is thus very conservative.

It is a fundamental prediction from evolutionary theory that convergence should continue at deeper times, and this is strongly supported as shown by the first four rows of results in Table 2, which use chloroplast genomes from deeper and deeper divergence times (column 4). This eliminates one simple model that allowed creation of ‘archetypes’ and limited evolution thereafter (see later discussion). Similarly, we find ancestral convergence with nuclear encoded sequences from vertebrates and invertebrates, and also with mitochondrial genomes from birds. Thus we have used chloroplast, nuclear, and mitochondrial DNA sequences, and from a wide variety of species. The times of divergence of the different datasets are estimated to vary from 80–700 millions of years ago (Mya) [18]–[22]. If we combine all 8 tests we get a *p* value of ≈ 2×10^−132^, and this is shown in the second to bottom row of Table 2.

The last two columns in Table 2 are control values where we compare the inferred ancestral sequence of one protein against the inferred ancestral sequence of a different protein. As expected, there is no tendency for these separate proteins to converge to similar sequences, making them good and effective controls. Indeed, the combined *p* value on the eight control datasets is *p* = 0.93172: indicating that the inferred ancestral scores are consistently below the average between-subgroups alignment score – again, our test is conservative.

The analyses establish that some form of ancestral convergence is occurring, and it is essential to explain the continued convergence as we go back to more distantly related organisms. Of course, such analyses by themselves cannot establish the mechanisms of evolutionary change (though the results are fully consistent with a stochastic mechanism, see also later).

## Discussion

Our test is based on the expectation that, under evolution, the ancestral sequence of one natural group of taxa will be more similar to the ancestral sequence of a second natural group of taxa, than to any sequence from the first group will be to any sequence from the second. In contrast, a variety of proposed non-evolutionary models either do not make this prediction, or require so many parameters that they cannot be said to make any testable predictions at all.

The basic results in Table 2 are overwhelming evidence that some form of ancestral convergence is occurring, and continues at deeper and deeper times. Individual tests have probabilities from 10^−6^ (for small numbers of genes) to 10^−44^ (for the larger number of genes in chloroplasts). Equally important, non-homologous controls show no tendency to converge (Table 2) – it is only homologous proteins that show ancestral convergence.

It is always possible to ‘design’ much more complex models where a separate decision by some unknown agent chooses/selects each amino acid change. With so many parameters the model is able to precisely mimic evolution (or indeed any other model); it has no discernible “signature” of its own. A minimum number of parameters for such a complex model can be determined by constructing, on the complete set of sequences of both subgroups, a variation on a maximum parsimony tree that allows single-residue insertions and deletions in addition to single-residue substitutions. This tree is an example of a Steiner tree [23] – a tree of minimal total edge length that connects a given set of points in a metric space, allowing for the introduction of new intermediate (ancestral) points as required. In this case, the distance between two sequences is given by the edit distance (the minimum number of single-residue insertions, deletions and substitutions [‘edits’] required to transform one sequence into the other).

The length of this tree is then by definition the minimum possible number of separate decisions, or equivalently free parameters, that a hypothetical external agent requires in order to produce the complete set of sequences, given any one of the sequences as a starting point. A lower bound for the length of a Steiner tree is given by half the length of a minimum spanning tree, which is a tree that connects all given points without introducing additional points [24]; minimum spanning trees can be computed efficiently. For the eudicot/monocot example, a minimum spanning tree requires 36,473 mutations to connect all 68 sequences, implying that we would need at least 36,473/2 = 18,237 free choices, each a separate parameter. Any suggestion that a model with such a huge number of parameters ‘explains’ the data is of course a serious violation of the scientific principle of selecting the simplest model.

Early (pre-Darwinian) biologists suggested several ideas as to the relationship of modern organisms, but a relevant one here is the ‘archetype’ model [25] that suggested that a number of ‘forms’ were originally created within high-level groups. For mammals say, one ‘form’ would have been a giant cat, which then independently evolved (or degenerated) into lions, tigers, leopards, panthers, cheetahs, etc. In our examples, this is tested (and eliminated) by demonstrating that successively deeper datasets continue to show ancestral convergence. In other words, we do not see a set of ‘archetype’ species originating at just one point in time – there is continuity in the evolutionary process. A qualification is that at the very deepest times we expect that information will be lost - this is a property of the Markov models used [26]. However, similar tests could be done at deeper times using measures of similarity of 3D structure. Indeed, it is important to note that modern methods of molecular biology now allow ancestral sequences to be synthesized, and the properties of the protein products of the ‘ancestral’ genes can be tested [27]. So there is now no doubt that these ancestral sequences do meet the functional requirements of the ancestors.

Perhaps, it is important for scientists to emphasize that by any scientific standard, evolution is simply inevitable. Good examples of continued evolution are RNA viruses, such as the influenza viruses; they just keep evolving from year to year – evolution in real time. New anti-viral immunisations are prepared for each northern hemisphere winter and for each southern hemisphere winter, and so on. Certainly, DNA-based organisms evolve more slowly; they have a lower mutation rate. But the inevitability of the process is there. “Stop the World, I want to get off”, was the title of a 1960s musical. We can neither stop the world (and get off), nor can we stop evolution. In the viral case there can also be recombination between RNA genes e.g. influenza, [28] or between sections of the genome (hepB) [29] – these recombinations are the equivalent of macroevolution (ref [28], Chap 5). We have already tested (and rejected) some ‘non-standard’ models for influenza evolution [30]. However, each gene (or section of the gene) should still converge, even if there is lateral gene transfer. Even though the fidelity of DNA copying is extraordinary - around 1 error in 10^9^–10^10^ nucleotides copied [31], no known organism can copy its DNA with absolute accuracy – thus there is always genetic diversity in natural populations.

So our conclusions are perhaps three-fold. Firstly we have provided a strong quantitative test rejecting a non-evolutionary model that amino acid sequences do not become more similar as we go back in time. Secondly, we have raised the problems of the number of parameters required of some alternatives, and finally we shift the requirement onto doubters to provide testable alternatives. On this third aspect, there does appear to also be a similar reaction from climate change advocates on placing responsibilities onto doubters [32]. Other aspects of evolution have been tested [33]–[35] and further aspects of evolution could be tested, perhaps especially the ‘random’ nature of mutations that occur without regard for any ‘need’ of the organism, but this is outside the scope of the present work. Indeed, there has always been excellent support for evolution from fossils and comparative morphology, and molecular data enables this to be quantitative. We can say that, as yet, no features of genomes have yet been found that are not understandable by ‘causes now in operation’ [4].

From the scientific point of view, there is no doubt that evolution has occurred, and there really were a continuous set of intermediates connecting individuals, populations, varieties, species, genera, families, etc. Nevertheless, as scientists we need to ensure that we have good quantitative tests available of all our favoured models. Given our results, we suggest that researchers need to be more assertive that evolution has both occurred, and continues to occur. It is essential that any person who does not accept the continuity of evolution puts forward alternative testable models. As we tell our first year undergraduates, ‘belief is the curse of the thinking class’.

Aligned datasets are available upon request from the authors. Alignments were carried out by BJZ, trees for each subset were inferred by WTJW, and DP designed the original study. All authors contributed to the final manuscript.
